# A Neuronal Network-Based Score Predicting Survival in Patients Undergoing Aortic Valve Intervention: The ABC-AS Score

**DOI:** 10.3390/jcm13133691

**Published:** 2024-06-25

**Authors:** Fabian Barbieri, Bernhard Erich Pfeifer, Thomas Senoner, Stephan Dobner, Philipp Spitaler, Severin Semsroth, Thomas Lambert, David Zweiker, Sabrina Barbara Neururer, Daniel Scherr, Albrecht Schmidt, Gudrun Maria Feuchtner, Uta Charlotte Hoppe, Agne Adukauskaite, Markus Reinthaler, Ulf Landmesser, Silvana Müller, Clemens Steinwender, Wolfgang Dichtl

**Affiliations:** 1 Department of Cardiology, Angiology and Intensive Care, Deutsches Herzzentrum der Charité, 12203 Berlin, Germany; markus.reinthaler@dhzc-charite.de (M.R.); ulf.landmesser@dhzc-charite.de (U.L.); 2 Department of Internal Medicine III, Medical University of Innsbruck, 6020 Innsbruck, Austria; thomas.senoner@tirol-kliniken.at (T.S.); philipp.spitaler@i-med.ac.at (P.S.); agne.adukauskaite@tirol-kliniken.at (A.A.); silvana.mueller@tirol-kliniken.at (S.M.); wolfgang.dichtl@tirol-kliniken.at (W.D.); 3 Institute of Clinical Epidemiology, Tirol Kliniken, 6020 Innsbruck, Austria; bernhard.pfeifer@tirol-kliniken.at (B.E.P.); s.neururer@tirol-kliniken.at (S.B.N.); 4 Division for Digital Medicine and Telehealth, University for Health Sciences, Medical Informatics and Technology (UMIT), 6060 Hall in Tirol, Austria; 5 Department of Cardiology, Inselspital, Bern University Hospital, University of Bern, 3010 Bern, Switzerland; stephandobner@me.com; 6 Department of Cardiology and Intensive Care, Clinic Ottakring, 1160 Vienna, Austria; davidzweiker@gmail.com; 7 University Clinic of Heart Surgery, Medical University of Innsbruck, Anichstrasse 35, 6020 Innsbruck, Austria; severin.semsroth@i-med.ac.at; 8 Department of Cardiology, Kepler University Hospital, Medical Faculty, Johannes Kepler University Linz, 4021 Linz, Austria; thomas.lambert@kepleruniklinikum.at (T.L.); clemens.steinwender@kepleruniklinikum.at (C.S.); 9 Department of Internal Medicine, Division of Cardiology, Medical University Graz, 8010 Graz, Austria; daniel.scherr@medunigraz.at (D.S.); albrecht.schmidt@medunigraz.at (A.S.); 10 University Clinic of Radiology, Medical University of Innsbruck, 6020 Innsbruck, Austria; gudrun.feuchtner@i-med.ac.at; 11 University Clinic of Internal Medicine II, Paracelsus Medical University, 5020 Salzburg, Austria; u.hoppe@salk.at; 12 Institute of Active Polymers and Berlin-Brandenburg Center for Regenerative Therapies, Helmholtz-Zentrum Hereon, 14513 Teltow, Germany; 13 DZHK (German Centre for Cardiovascular Research), Partner Site Berlin, 10785 Berlin, Germany; 14 Berlin Institute of Health (BIH), 10178 Berlin, Germany

**Keywords:** biomarker, risk score, artificial intelligence, risk prediction model, aortic valve, aortic stenosis, aortic valve replacement, transcatheter aortic valve replacement, transcatheter aortic valve implantation

## Abstract

**Background**: Despite being the most commonly performed valvular intervention, risk prediction for aortic valve replacement in patients with severe aortic stenosis by currently used risk scores remains challenging. The study aim was to develop a biomarker-based risk score by means of a neuronal network. **Methods**: In this multicenter study, 3595 patients were divided into test and validation cohorts (70% to 30%) by random allocation. Input variables to develop the ABC-AS score were age, the cardiac biomarker high-sensitivity troponin T, and a patient history of cardiac decompensation. The validation cohort was used to verify the scores’ value and for comparison with the Society of Thoracic Surgery Predictive Risk of Operative Mortality score. **Results**: Receiver operating curves demonstrated an improvement in prediction by using the ABC-AS score compared to the Society of Thoracic Surgery Predictive Risk of Operative Mortality (STS prom) score. Although the difference in predicting cardiovascular mortality was most notable at 30-day follow-up (area under the curve of 0.922 versus 0.678), ABC-AS also performed better in overall follow-up (0.839 versus 0.699). Furthermore, univariate analysis of ABC-AS tertiles yielded highly significant differences for all-cause (*p* < 0.0001) and cardiovascular mortality (*p* < 0.0001). Head-to-head comparison between both risk scores in a multivariable cox regression model underlined the potential of the ABC-AS score (HR per z-unit 2.633 (95% CI 2.156–3.216), *p* < 0.0001), while the STS prom score failed to reach statistical significance (*p* = 0.226). **Conclusions**: The newly developed ABC-AS score is an improved risk stratification tool to predict cardiovascular outcomes for patients undergoing aortic valve intervention.

## 1. Introduction

Aortic stenosis (AS) represents the most common type of valvular heart disease in high-income countries [1]. For patients with severe, symptomatic AS, the therapy of choice remains valve intervention, either by transcatheter aortic valve implantation (TAVI) or conventional surgical aortic valve replacement (SAVR) [2]. Accordingly, there is a high demand for precise risk stratification tools to predict procedural success and postoperative mortality.

Currently, the most commonly applied risk scores for the prediction of postoperative mortality are the Society of Thoracic Surgery Predictive Risk of Operative Mortality (STS prom) and the European System for Cardiac Operative Risk Evaluation II (EuroSCORE II), an updated form of the logistic EuroSCORE, which was first described in 2012 [3,4,5]. Both scores have been primarily developed to predict perioperative mortality, yet they have also been proposed to prognosticate long-term survival [6,7]. While most comparative trials report an equivalence of the EuroSCORE II and the STS prom score, their ability to accurately predict clinical outcomes is modest [8,9,10]. In daily clinical practice, the STS prom is most widely used, as it was used to inform about patient risk in all three PARTNER trials, which compared TAVI to standard medical therapy in patients with severe aortic stenosis [11,12,13]. The calculation of the STS prom risk score, though, remains bothersome due to the vast amounts of parameters included. Simpler and more accurate risk stratification tools, similar to the recently proposed ABC (age, biomarkers, and clinical history) score for the prediction of stroke and death in atrial fibrillation [14,15,16], are required to help direct treatment pathways in patients with severe AS requiring valve intervention. Recent investigations have suggested a role for well-established and readily available cardiac biomarkers, such as high-sensitivity troponin T (hsTnT) and N-terminal pro-brain natriuretic peptide (NT-proBNP), to predict clinical outcomes in patients with severe AS irrespective of the interventional therapy chosen. Although cardiac biomarkers, a centerpiece in personalized medicine, have shown these promising results, they have yet to be included in stratification tools [3,4,5,17,18,19,20,21,22]. Furthermore, artificial intelligence, such as deep learning algorithms, may help in improving the accuracy of risk prediction modeling, which is particularly crucial for cardiovascular disease management [23,24]. Using this technology, we developed a cardiac-biomarker-based risk score, consisting of hsTnT, age, and patient history of cardiac decompensation. The aim of the current study was to evaluate the potential of the ABC-AS score to accurately predict perioperative and clinical outcomes in patients with severe AS undergoing aortic valve intervention.

## 2. Materials and Methods

### 2.1. Study Design

The investigation represents a post hoc analysis of the Tyrolean Aortic Stenosis Study-2 (TASS-2), a retrospective study initiated to evaluate the predictive value of hsTnT to predict postprocedural survival. The collaboration was established at the Medical University of Innsbruck (MUI) in 2013 and included three other major Austrian university hospitals [Medical University Graz (MUG), Johannes Kepler University Linz (KUK), and Paracelsus Medical University Salzburg (PMU)]. All patients diagnosed with severe AS undergoing valve intervention between 2007 and 2017 were screened for study inclusion. Data collection was performed in accordance with regulations set forth by institutional review boards and was limited to TAVI patients at the MUG and PMU. The trial was approved by institutional review boards/independent ethics committees and registered at ClinicalTrials.gov (NCT02448485). This study followed the TRIPOD Statement for development and reporting of our predictive model [25].

### 2.2. Study Population

Consecutive patients referred for evaluation of aortic valve intervention were screened for study participation. The only inclusion criterion was the presence of severe AS according to current guidelines [2], requiring aortic valve intervention by either SAVR or TAVI, respectively. Previous endocarditis and/or aortic valve replacement due to non-severe AS, redo aortic valve intervention, Ross procedure surgery, sub-valvular severe AS, acute coronary syndrome or cardiopulmonary resuscitation within two months prior to valve intervention, conversion of TAVI to SAVR during the procedure, balloon aortic valvuloplasty for bridging, unknown coronary anatomy, and other valve pathologies considered as the main clinical problem were the predefined exclusion criteria [17,18,26]. Patients with missing variables to calculate the ABC-AS score were excluded.

### 2.3. Data Collection

Clinical data, including the STS prom score, were collected from electronic patient records at participating institutions. Age (in years) was calculated by using the date of procedure, while only a single preprocedural measurement of hsTnT was taken into account (either at admission or referral from another hospital). Cardiac decompensation was defined as history of at least one episode of acute worsening of heart failure symptoms requiring hospitalization or emergency department visits due to acute heart failure requiring intravenous diuretics (binary). Clinical outcome data (cardiovascular and all-cause mortality) were provided by Statistik Austria, the national statistical office, in the form of ICD-10 codes. If the cause of death was uncertain, patients were excluded from the cardiovascular mortality analysis.

### 2.4. Artificial Intelligence Model

A labeled dataset and their corresponding desired outputs and target values were prepared. Patients were divided into training and validation cohorts by random allocation and by applying a 70% to 30% rule. The type and structure of the neural network was chosen based on the problem at hand. The network’s weights and biases were initialized randomly. The computed output was compared to the desired output for each input sample, and an error metric (loss function) was calculated (MSE). For adjusting weights, the error was propagated backwards through the network in order to minimize the overall error. The model was iterated and stopped at convergence criterion. The predictive model was trained by using overall all-cause mortality as the endpoint. For parameterization, a logistic function was used, as a rectified linear unit was less successful in training our overall model. The maximum number of iteration steps, in case the system did not converge, was set to 10 × 10^7^ steps. The model consisted of 4 input channels and 1 output channel, while 3 hidden channels were used, holding 5 neurons at each level. All tested models underwent a validation process for model integrity. Different settings were investigated but did not improve the results or overall behavior. The output variable was the probability of occurrence of an event (herein, all-cause mortality reported values between 0 and 1 to display probability). With this approach, which is widely used in the artificial intelligence community, a good setting for our described problem was found [27,28,29,30,31,32].

### 2.5. Statistics

Continuous variables are displayed as median (interquartile range), whereas categorical variables are presented as number of patients (percentage). Distribution of continuous variables was assessed by inspection of histograms and the Shapiro–Wilk test. Calculated ABC-AS scores were separated into three tertiles for univariable survival analyses, which were assessed by Kaplan–Meier curves and applying the log-rank test. Significance of differences between continuous variables was assessed either by *t*-tests and analysis of variances (ANOVA) or Mann–Whitney U and Kruskal–Wallis tests, according to their distribution. Differences between categorical variables were calculated by the Chi square test. Receiver operating curves (ROC) with their respective area under the curve (AUC) were used to estimate the predictive value of the ABC-AS and STS prom score. Both variables were subsequently used in a cox proportional hazards regression model after z-standardization to allow direct comparison. Statistics were performed with R Studio version 4.2.2 (R Studio Inc., Boston, MA, USA) and IBM SPSS version 24 (IBM Corporation, Armonk, NY, USA), and graphics were designed using GraphPad PRISM, version 5 (GraphPad Software, Inc., La Jolla, CA, USA). The nnet package, version 4.2.1, was used to establish the neuronal network in R Studio. Two-sided *p*-values of ≤0.05 were considered significant.

## 3. Results

### 3.1. Study Population

**Figure 1:**
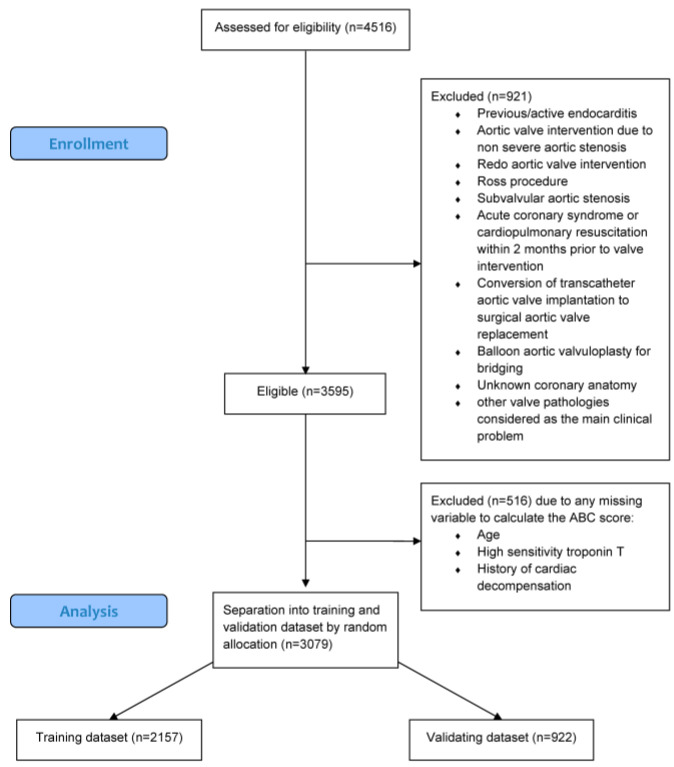
Flow diagram.

Overall, 4516 patients referred for aortic valve intervention were screened (Figure 1). Nine hundred and twenty-one patients (20.4%) were excluded by predefined criteria. Accordingly, 3595 patients were enrolled into the study (MUI: 1624 patients, KUK: 1091 patients, MUG: 556 patients, and PMU: 324 patients). Five hundred and sixteen patients (14.4%) were excluded due to missing variables to calculate the ABC-AS score (Figure 1). Remaining patients (*n* = 3079) were divided into a training (*n* = 2157) and a validation cohort (*n* = 922, Figure 1). Considering the uncertainty about the cause of death in a single patient, he was excluded from the analysis of cardiovascular mortality.

### 3.2. Baseline Characteristics

Baseline characteristics of the training and validation cohort are described in Table 1. The validating database, which was also used for further analysis, displayed a median follow-up of 1.5 years (interquartile range: 0.5–3.0). SAVR was performed in 543 (58.9%) patients, and TAVI in 379 (41.1%). Median STS prom score was 2.54% (1.57–3.80). Overall, all-cause mortality occurred in 166 (18.0%) patients, out of which 87 (9.4%) were due to cardiovascular causes. Thirty-day all-cause mortality was observed in 28 (3.0%) patients, while the number increased steadily (90-day mortality: 47, 5.1%; 365-day mortality: 66, 7.2%) throughout extension of follow-up. Cardiovascular mortality increased in a similar manner (30-day cardiovascular mortality: 18, 2.0%; 90-day mortality: 30, 3.3%; 365-day mortality: 42, 4.6%). Overall, comparison of baseline characteristics between the training and validation cohorts showed that the validation cohort more frequently suffered from comorbidities. In particular, patients were more often found to have a history of cardiac decompensation (*p* = 0.002), arterial hypertension (*p* < 0.001), hypercholesterolemia (*p* < 0.001), chronic obstructive pulmonary disease (*p* = 0.003), carotid stenosis (*p* = 0.001), and obstructive coronary artery disease (*p* = 0.001). On the other hand, patients of the training cohort displayed higher aortic valve mean gradients (*p* < 0.001) and a lower stroke volume index (*p* < 0.001). There were no significant differences regarding hsTnT, NT-proBNP, renal function, left ventricular ejection fraction, prescription of heart failure medication, and the procedure conducted (TAVI or SAVR). 

Based on obtained values of the ABC-AS score, the validating dataset was separated into three tertiles. Their baseline characteristics are displayed in Table 2. Patients in the third tertile were older (1st: 69 years (61–75), 2nd: 78 (73–81), 3rd: 82 (77–85)), yielded the highest preprocedural hsTnT plasma levels (1st: 9.7 (6.0–14.9), 2nd: 19.0 (13.0–26.7), 3rd: 31.0 (20.0–53.0)), and more often presented with a history of cardiac decompensation (1st: 20 (6.5%), 2nd: 64 (20.8%), 3rd: 131 (42.7%)). The third tertile was also found to have a smaller aortic valve area (1st: 0.70 cm^2^ (0.60–0.88), 2nd: 0.70 (0.55–0.80), 3rd: 0.60 (0.50–0.80), *p* < 0.0001), which did not change after indexation with body surface area by the DuBois formula (*p* < 0.0001). Furthermore, patients in the third tertile suffered more frequently from significant coronary artery disease (*p* < 0.0001), left ventricular ejection fraction impairment (*p* < 0.0001), atrial fibrillation (*p* < 0.0001), and arterial hypertension (*p* = 0.029), and were less likely to be asymptomatic (*p* < 0.0001). NT-proBNP plasma levels (*p* < 0.0001) as well as the STS prom score (*p* < 0.0001) increased, whereas estimated glomerular filtration rate (*p* < 0.0001) decreased throughout the tertiles. Similarly, the proportion of TAVI as the procedure of choice rose from the first to the third tertile (1st: 34 (11.0%), 2nd: 141 (45.9%), 3rd: 204 (66.4%)).

### 3.3. Receiver Operating Curves to Evaluate the Predictive Value

**Figure 2:**
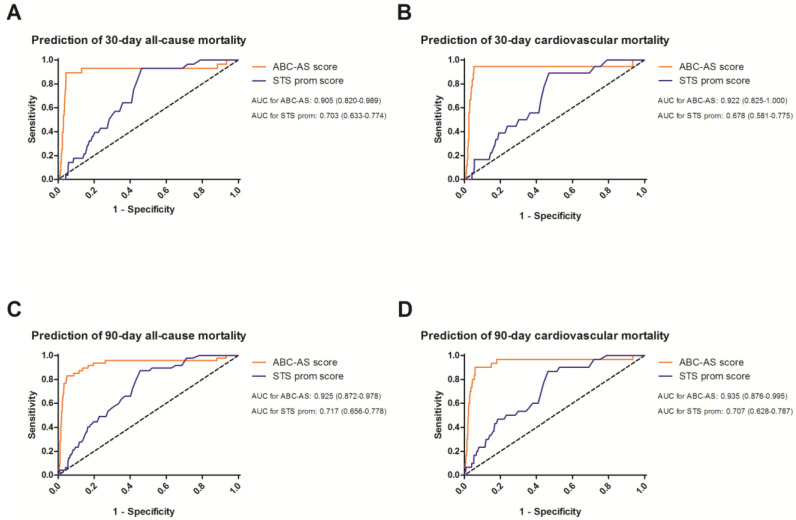
Receiver operating curves for the prediction of 30-day all-cause (**A**) and cardiovascular mortality (**B**), as well as 365-day all-cause (**C**) and cardiovascular mortality (**D**). Area under the curve (AUC) is presented for the ABC-AS and Society of Thoracic Surgery Predictive Risk of Operative Mortality (STS prom) score.

**Figure 3:**
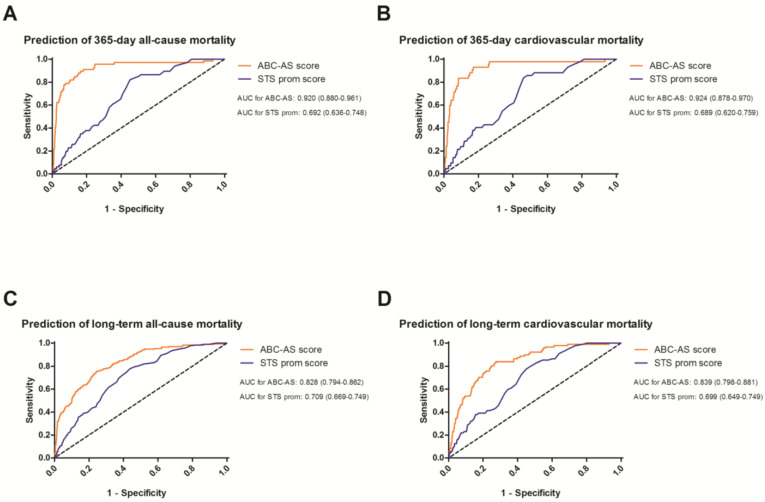
Receiver operating curves for the prediction of 365-day all-cause (**A**) and cardiovascular mortality (**B**), as well as long-term all-cause (**C**) and cardiovascular mortality (**D**). Area under the curve (AUC) is presented for the ABC-AS and Society of Thoracic Surgery Predictive Risk of Operative Mortality (STS prom) score.

The assessment of both scores and their corresponding predictive values is shown by ROC curves (Figure 2 and Figure 3). Especially during short-term follow-up (Figure 2), the ABC-AS score was able to outperform the predictability of the STS prom score. For 30-day all-cause mortality, the AUC of the ABC-AS score (AUC: 0.905 (95% confidence interval (CI): 0.820–0.989)) showed an improved predictability compared to the STS prom score (0.703 (0.633–0.774)). Similar results were obtained when comparing the ROC curves of 30-day cardiovascular mortality (0.922 (0.825–1.000) versus 0.678 (0.581–0.775)), 90-day all-cause mortality (0.925 (0.872–0.978) versus 0.717 (0.656–0.778)), and 90-day cardiovascular mortality (0.935 (0.876–0.995) versus 0.707 (0.628–0.787)). 

The predictability of the ABC-AS score continued to outperform the STS prom score for 365-day all-cause (0.920 (0.880–0.961) versus 0.692 (0.636–0.748)) and cardiovascular mortality (0.924 (0.878–0.970)) versus (0.689 (0.620–0.759)). The difference between both scores was still present while utilizing complete follow-up, yet the margin was found to be smaller (all-cause mortality: 0.828 (0.794–0.862) versus 0.709 (0.669–0.749); cardiovascular mortality: 0.839 (0.798–0.881) versus 0.699 (0.649–0.749)), respectively (Figure 3).

### 3.4. Univariate Analysis with Kaplan–Meier Curves

**Figure 4:**
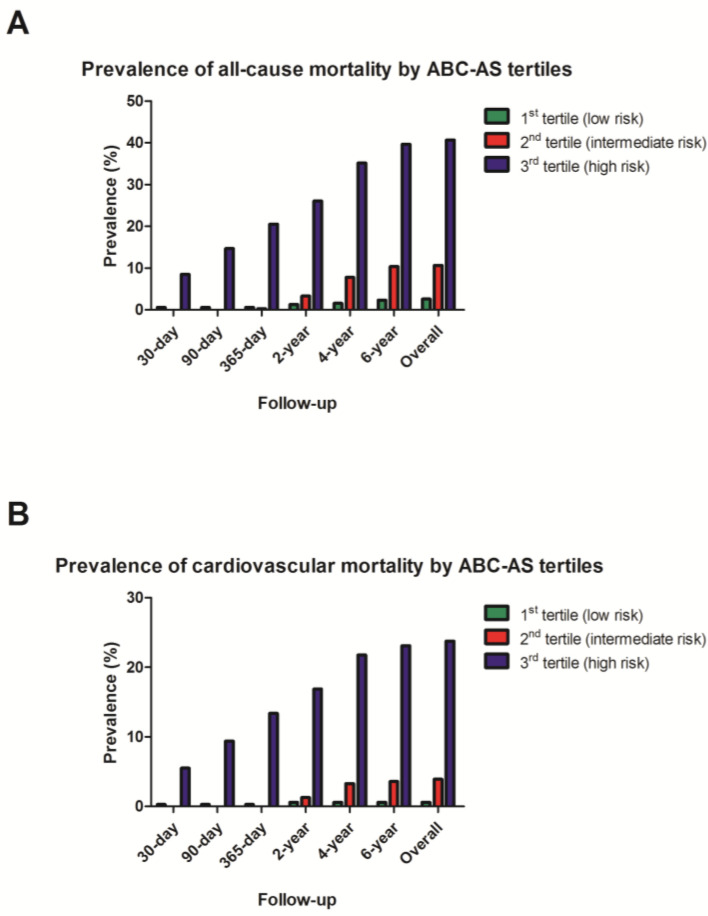
Prevalence of all-cause (**A**) and cardiovascular mortality (**B**) by ABC-AS tertiles during follow-up.

**Figure 5:**
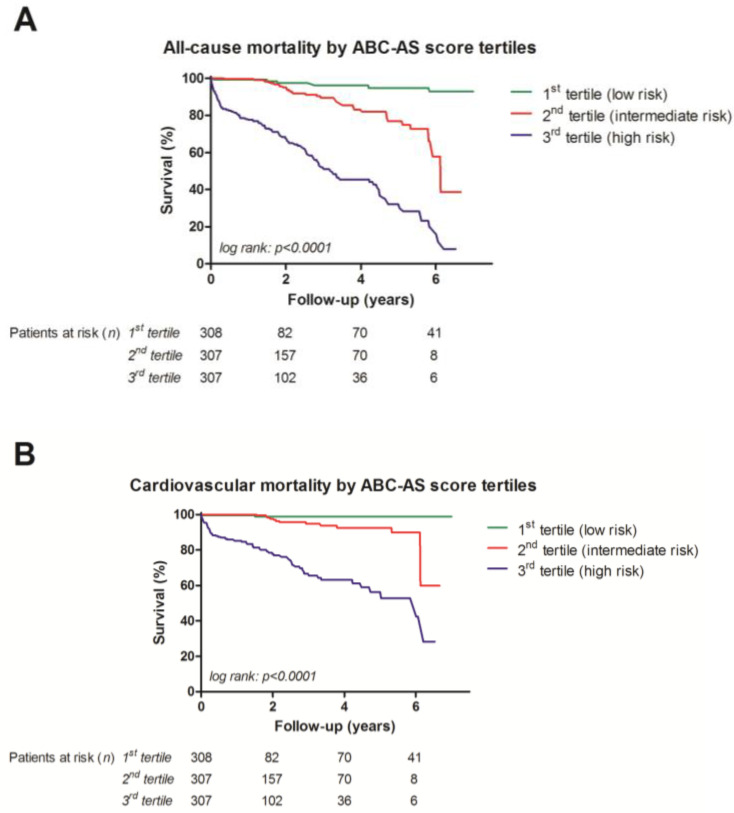
Kaplan–Meier curves showing differences between the three ABC-AS score tertiles for all-cause (**A**) and cardiovascular mortality (**B**).

Prevalence of cardiovascular and all-cause mortality, separated by the ABC-AS tertiles, is displayed in Figure 4. Univariate analysis (Figure 5) of ABC-AS score tertiles by using the Kaplan–Meier method yielded highly significant differences for all-cause (*p* < 0.0001) and cardiovascular mortality (*p* < 0.0001). Subgroup analysis by stratification for type of procedure yielded similar results. In patients undergoing TAVI, the log-rank test remained highly statistically significant for both all-cause (*p* < 0.0001) and cardiovascular mortality (*p* < 0.0001). Furthermore, ABC-AS score tertiles were also able to predict all-cause (*p* < 0.0001) and cardiovascular mortality (*p* < 0.0001) in the surgical subgroup.

### 3.5. Multivariate Analysis for Direct Comparison

Direct comparison of the ABC-AS and STS prom scores was conducted by calculating cox proportional hazards regression models after standardization. Regarding all-cause mortality, the ABC-AS score remained highly statistically significant (hazard ratio (HR) per z-unit 3.085 (95% CI 2.533–3.757), *p* < 0.0001), whereas the STS prom score was unable to reach significance (HR per z-unit 1.144 (95% CI 0.952–1.376), *p* = 0.151). Similar results were obtained in the second model on cardiovascular mortality. The ABC-AS score remained statistically significant (HR per z-unit 2.633 (95% CI 2.156–3.216), *p* < 0.0001), while the STS prom score failed to reach the significance margin (HR per z-unit 1.144 (95% CI 0.920–1.423), *p* = 0.226).

## 4. Discussion

Using the established predictive value of routinely available biomarkers, this study proposed a novel artificial-intelligence-based risk score to prognosticate postoperative mortality in patients undergoing valve intervention due to AS. The ABC-AS score, a composition of age, biomarker (hsTnT), and cardiovascular history (prior cardiac decompensation), is the first biomarker-based risk score and consistently outperformed the guidelines recommended by the STS prom score in the presented analysis. It was also able to maintain its predictive value irrespective of the procedure chosen (TAVI or SAVR), as indicated by subgroup analysis. Furthermore, the ABC-AS score limits the shortcomings of the currently used risk scores, which are mostly meant to predict short-term postoperative outcomes. This may also be of importance for future trials assessing long-term differences. A valid risk score, which reliably predicts short-, mid-, and long-term survival, would allow more definitive results in comparative trials between TAVI and SAVR, and ensure that long-term survival is not affected by preoperative differences not displayed in the currently used risk scores. 

The centerpiece of our score is the utilization of hsTnT. Besides its well-known role in predicting critical ischemia following the kinetics of an acute-phase marker (“rise and fall” due to necrosis), it is also regularly released due to turnover by secretory autophagy to maintain homeostasis [33,34,35]. This phenomenon may be upregulated due to cell wounding by increased pre- or after-load, such as, for example, in the presence of severe AS, causing myocardial strain, hence leading to a stable increase in hsTnT serum plasma levels [36]. Recently, a study by Perry et al. showed the strong association between myocardial strain, assessed by echocardiography, and hsTnT [37]. While the increase shows the sheer stress to the myocardium, elimination of these steadily increased hsTnT plasma levels seems to play an important role in the predictive value as well. Although previous studies have demonstrated that both the hepatic and renal systems are responsible for hsTnT clearance, higher concentrations of hsTnT are still mainly eliminated by the hepatic system. At lower levels, though, the renal system’s contribution to the elimination increases [38,39]. Chronic kidney diseases, on the other hand, reduce the continuous elimination of hsTnT, which then increases plasma levels and, therefore, reflects the disease in its own way. 

Apart from myocardial strain and renal function, hsTnT plasma levels are also strongly influenced by the presence of cardiac amyloidosis and, therefore, also reflect the independent risk of morbidity and mortality of a disease currently not covered by risk scores [40]. Especially, the subtype of transthyretin amyloidosis is an underdiagnosed comorbidity found in up to 15% in the overall cohort of AS, and in up to 30% of patients with low-flow, low-gradient AS [41]. 

Overall, many factors and comorbidities are reflected by hsTnT. This of course also holds true for the proposed ABC-AS score, which showed an increase in morbidity from tertile to tertile. Implementation of biomarkers in already available risk scores would also seem reasonable. However, another increase in variables would further complicate these scores. Instead, an easy to remember and, namely, in-line risk stratification tool, seems rather appropriate. It requires less parameters and may, therefore, be calculated quickly in daily clinical routine. 

Comparing our results in c-statistics for the STS prom to available data in the literature, similar predictive values were observed, thus underlining the data quality and the potential of our ABC-AS score. Internal and external validation datasets reported an AUC ranging from 0.67 to 0.78 for 30-day mortality and 0.73 to 0.77 for mid- to longer-term mortality [4,6,9,10]. Results for the EUROSCORE II were comparable, achieving an AUC ranging from 0.65 to 0.81 for 30-day mortality and 0.73 to 0.77 for mid- to longer-term mortality [5,7,9,10].

A definitive strength of our analysis, which is often found to be a weakness for reproducibility of neuronal networks, was the relatively small risk for data quality bias. Measurements of hsTnT were routinely conducted at hospital admission, while age is easily assessed by calculation. Endpoints were provided by the Austrian National Statistics department, assuring high data quality. An online calculator is available at: https://surviral.at.

## 5. Limitations

A major limitation is the retrospective data collection, as well as the cohort study design with its respective flaws and bias. Considering the retrospective design, a unified definition of cardiac decompensation was impossible and was mainly based on physicians’ discretion. Differences in baseline characteristics between the training and validation cohorts were mainly due to taking samples, as random allocation was utilized. The proposed ABC-AS score is currently only validated by an internal dataset and requires external validation before its use should be recommended in daily clinical routine. Furthermore, the dataset used for training of the neuronal network can be considered as rather small in the world of artificial intelligence. Lastly, there is no mathematical method to define the optimal settings for the development of a neuronal network. Nonetheless, all efforts were made to comply with the recently published guidelines and quality criteria for artificial-intelligence-based prediction models. Generally, there are various metrics for evaluating the performance of an AI model, depending on the type of problem. Examples are accuracy, precision, recall, and F1-score for classification tasks, RMSE (root mean squared error) and MAE (mean absolute error) for regression tasks, and AUC-ROC. The ROC curve was mainly used in our approach, as it provides a clear representation of the performance of the presented AI model [27,28,29,30,31,32].

## Figures and Tables

**Table 1:** Baseline characteristics of the training and validation cohorts.

Variable	Training Cohort (*n* = 2157)	Validation Cohort (*n* = 922)	*p*-Value
Age (years)	76 (70–82)	77 (70–82)	n.s.
Gender (female), *n* (%)	1036 (48.0%)	436 (47.3%)	n.s.
Height (cm)	168 (160–174)	168 (162–174)	n.s.
Weight (kg)	75 (65–85)	75 (65–85)	n.s.
Body surface area (DuBois, m^2^)	1.84 (1.69–1.98)	1.84 (1.71–2.00)	n.s.
History of cardiac decompensation, *n* (%)	319 (18.3)	215 (23.3)	0.002
Stable heart failure symptoms, *n* (%)	1681 (80.2)	528 (57.8)	<0.001
Stable angina pectoris, *n* (%)	804 (38.4)	305 (33.4)	0.009
History of syncope, *n* (%)	204 (11.7)	125 (13.7)	n.s.
Asymptomatic, *n* (%)	60 (3.4)	29 (3.1)	n.s.
Arterial hypertension, *n* (%)	1693 (78.6)	798 (86.6)	<0.001
Diabetes mellitus, *n* (%)	515 (23.9)	218 (23.6)	n.s.
Hypercholesterolemia, *n* (%)	939 (53.2)	609 (66.1)	<0.001
Nicotine			n.s.
Active smoker, *n* (%)	127 (7.3)	68 (8.4)	
Former smoker, *n* (%)	274 (15.7)	122 (15.1)	
History of stroke, *n* (%)	196 (9.1)	89 (9.7)	n.s.
Atrial fibrillation			n.s.
Paroxysmal, *n* (%)	255 (11.8)	99 (10.7)	
Persistent/permanent, *n* (%)	351 (16.3)	148 (16.1)	
Chronic obstructive pulmonary disease, *n* (%)	304 (14.1)	168 (18.3)	0.003
Carotid stenosis (≥50%), *n* (%)	115 (5.3)	88 (9.7)	<0.001
Coronary artery disease			0.001
No significant coronary artery disease, *n* (%)	1374 (63.7)	535 (58.1)	
1-vessel disease, *n* (%)	343 (15.9)	183 (19.9)	
2-vessel disease, *n* (%)	189 (8.8)	94 (10.2)	
3-vessel disease, *n* (%)	173 (8.0)	91 (9.9)	
Left main disease, *n* (%)	78 (3.6)	18 (2.0)	
Left ventricular ejection fraction			n.s.
>50%, *n* (%)	1558 (75.3)	701 (77.9)	
35–50%, *n* (%)	430 (20.8)	171 (19.0)	
<35%, *n* (%)	80 (3.9)	28 (3.1)	
Aortic valve mean pressure gradient (mmHg)	50 (41–60)	46 (40–57)	<0.001
Aortic valve area (cm^2^)	0.70 (0.55–0.80)	0.70 (0.55–0.80)	n.s.
Indexed aortic valve area (DuBois, cm^2^/m^2^)	0.37 (0.30–0.44)	0.36 (0.30–0.44)	n.s.
Stroke volume index (DuBois, mL/m^2^)	29.0 (24.0–34.0)	33.0 (27.0–40.0)	<0.001
Total cholesterol (mg/dL)	174 (142–207)	174 (142–203)	n.s.
LDL cholesterol (mg/dL)	102 (79–129)	96 (71–124)	<0.001
HDL cholesterol (mg/dL)	54 (43–66)	56 (45–70)	<0.001
Triglycerides (mg/dL)	99 (71–139)	104 (80–141)	0.001
High-sensitivity troponin T (ng/L)	18.0 (11.0–33.4)	17.8 (10.7–30.0)	n.s.
N-terminal pro-brain natriuretic peptide (ng/L)	1284 (512–3005)	1345 (458–3443)	n.s.
Creatinine (mg/dL)	1.00 (0.80–1.20)	1.00 (0.85–1.20)	0.004
Estimated glomerular filtration rate (mL/min/1.73 m^2^)	81.0 (66.9–92.9)	80.9 (62.5–91.9)	n.s.
STS Predicted Risk of Mortality (%)	2.56 (1.61–4.01)	2.54 (1.57–3.80)	n.s.
Medication			
Betablocker, *n* (%)	1064 (50.6)	487 (53.2)	n.s.
Calcium channel blocker, *n* (%)	349 (19.8)	195 (21.4)	n.s.
ACE inhibitor/ARB/ARNI, *n* (%)	1266 (60.2)	564 (61.6)	n.s.
Acetyl salycilyc acid, *n* (%)	1149 (57.1)	580 (63.3)	0.001
P2Y12 antagonists, *n* (%)	375 (17.8)	90 (9.8)	<0.001
Vitamin K antagonist, *n* (%)	369 (17.5)	130 (14.2)	0.023
Direct oral anticoagulants, *n* (%)	119 (5.9)	86 (9.4)	0.001
Hydrochlorothiazide, *n* (%)	499 (28.4)	234 (25.6)	n.s.
Loop diuretic, *n* (%)	768 (38.1)	390 (42.6)	0.021
Statin, *n* (%)	1085 (53.9)	552 (60.3)	0.001
Aldosterone antagonist, *n* (%)	260 (12.4)	93 (10.2)	n.s.
Insulin, *n* (%)	77 (4.4)	48 (5.3)	n.s.

Numbers are presented as median (interquartile range) or number of patients (percentage). Abbreviations: ACE, angiotensin-converting enzyme; ARB, angiotensin receptor blocker; ARNI, angiotensin receptor neprilysin inhibitor; STS, Society of Thoracic Surgeons.

**Table 2:** Baseline characteristics of the validation cohort separated into ABC-AS tertiles.

Variable	1st Tertile (*n* = 308)	2nd Tertile (*n* = 307)	3rd Tertile (*n* = 307)
Age (years)	69 (61–75)	78 (73–81)	82 (77–85)
Gender (female), *n* (%)	140 (45.5%)	160 (52.1%)	136 (44.3%)
Height (cm)	168 (162–175)	168 (162–173)	168 (160–174)
Weight (kg)	78 (68–88)	75 (65–85)	71 (63–83)
Body surface area (DuBois, m^2^)	1.88 (1.76–2.03)	1.85 (1.71–1.99)	1.79 (1.68–1.96)
History of cardiac decompensation, *n* (%)	20 (6.5)	64 (20.8)	131 (42.7)
Stable heart failure symptoms, *n* (%)	198 (64.3)	191 (62.2)	139 (45.3)
Stable angina pectoris, *n* (%)	127 (41.2)	94 (30.6)	84 (27.4)
History of syncope, *n* (%)	39 (12.7)	46 (15.0)	40 (13.0)
Asymptomatic, *n* (%)	21 (6.8)	5 (1.6)	3 (1.0)
Arterial hypertension, *n* (%)	254 (82.5)	270 (87.9)	274 (89.3)
Diabetes mellitus, *n* (%)	58 (18.8)	80 (26.1)	80 (26.1)
Hypercholesterolemia, *n* (%)	224 (72.7)	205 (66.8)	180 (58.6)
Nicotine			
Active smoker, *n* (%)	24 (7.8)	22 (7.2)	22 (7.2)
Former smoker, *n* (%)	63 (20.5)	33 (10.7)	26 (8.5)
History of stroke, *n* (%)	22 (7.1)	32 (10.4)	35 (11.4)
Atrial fibrillation			
Paroxysmal, *n* (%)	24 (7.8)	33 (10.7)	42 (13.7)
Persistent/permanent, *n* (%)	27 (8.7)	46 (15.0)	75 (24.5)
Chronic obstructive pulmonary disease, *n* (%)	45 (14.6)	60 (19.5)	63 (20.5)
Carotid stenosis (≥50%), *n* (%)	22 (7.2)	28 (9.4)	38 (12.6)
Coronary artery disease			
No significant coronary artery disease, *n* (%)	197 (64.0)	194 (63.2)	144 (46.9)
1-vessel disease, *n* (%)	53 (17.2)	60 (19.5)	70 (22.8)
2-vessel disease, *n* (%)	26 (8.4)	28 (9.1)	40 (13.0)
3-vessel disease, *n* (%)	27 (8.8)	23 (7.5)	41 (13.4)
Left main disease, *n* (%)	5 (1.6)	2 (0.7)	11 (3.6)
Left ventricular ejection fraction			
>50%, *n* (%)	268 (87.0)	233 (75.9)	200 (65.1)
35–50%, *n* (%)	34 (11.0)	61 (19.9)	76 (24.8)
<35%, *n* (%)	2 (0.6)	7 (2.3)	19 (6.2)
Aortic valve mean pressure gradient (mmHg)	45 (40–57)	47 (41–58)	46 (40–58)
Aortic valve area (cm^2^)	0.70 (0.60–0.88)	0.70 (0.55–0.80)	0.60 (0.50–0.80)
Indexed aortic valve area (DuBois, cm^2^/m^2^)	0.38 (0.32–0.45)	0.36 (0.30–0.43)	0.35 (0.28–0.42)
Stroke volume index (DuBois, mL/m^2^)	33.0 (27.0–40.0)	35.0 (26.0–42.0)	31.0 (25.0–38.3)
Total cholesterol (mg/dL)	180 (148–213)	171 (140–197)	171 (135–198)
LDL cholesterol (mg/dL)	108 (84–135)	93 (69–118)	88 (63–115)
HDL cholesterol (mg/dL)	56 (48–69)	56 (47–70)	56 (43–70)
Triglycerides (mg/dL)	106 (81–145)	106 (78–144)	101 (79–134)
High-sensitivity troponin T (ng/L)	9.7 (6.0–14.9)	19.0 (13.0–26.7)	31.0 (20.0–53.0)
N-terminal pro-brain natriuretic peptide (ng/L)	449 (215–1270)	1517 (667–3239)	2827 (1213–6015)
Creatinine (mg/dL)	0.93 (0.80–1.05)	1.00 (0.84–1.20)	1.16 (0.96–1.51)
Estimated glomerular filtration rate (mL/min/1.73 m^2^)	90.4 (81.9–101.9)	78.3 (61.9–87.9)	66.9 (48.7–82.1)
STS Predicted Risk of Mortality (%)	1.50 (0.95–2.26)	2.46 (1.79–3.51)	3.70 (2.84–5.30)
Medication			
Betablocker, *n* (%)	147 (47.7)	167 (54.4)	173 (56.4)
Calcium channel blocker, *n* (%)	63 (20.5)	68 (22.1)	64 (20.8)
ACE inhibitor/ARB/ARNI, *n* (%)	187 (60.7)	190 (61.9)	187 (60.9)
Acetyl salycilyc acid, *n* (%)	195 (63.3)	197 (64.2)	188 (61.2)
P2Y12 antagonists, *n* (%)	16 (5.2)	29 (9.4)	45 (14.7)
Vitamin K antagonist, *n* (%)	28 (9.1)	41(13.4)	61 (19.9)
Direct oral anticoagulants, *n* (%)	15 (4.9)	31 (10.1)	40 (13.0)
Hydrochlorothiazide, *n* (%)	79 (25.6)	84 (27.4)	71 (23.1)
Loop diuretic, *n* (%)	57 (18.5)	140 (45.6)	193 (62.9)
Statin, *n* (%)	194 (63.0)	192 (62.5)	166 (54.1)
Aldosterone antagonist, *n* (%)	14 (4.5)	33 (10.7)	46 (15.0)
Insulin, *n* (%)	11 (3.6)	17 (5.5)	20 (6.5)

Numbers are presented as median (interquartile range) or number of patients (percentage). Abbreviations: ACE, angiotensin-converting enzyme; ARB, angiotensin receptor blocker; ARNI, angiotensin receptor neprilysin inhibitor; STS, Society of Thoracic Surgeons.

## Data Availability

The data presented in this study are available upon request from the corresponding author due to privacy and ethical restrictions.
